# Nanodiamonds enable femtosecond-processed ultrathin glass as a hybrid quantum sensor

**DOI:** 10.1038/s41598-023-30689-7

**Published:** 2023-04-18

**Authors:** Bhavesh K. Dadhich, Biswajit Panda, Mehra S. Sidhu, Kamal P. Singh

**Affiliations:** 1 Indian Institute of Science Education and Research Mohali, Sector 81, Mohali, 140306 India; 2 Present Address: Dept. of Soil Science, Punjab Agricultural University, Ludhiana, India

**Keywords:** Engineering, Materials science, Nanoscience and technology, Optics and photonics

## Abstract

The quantum properties of fluorescent nanodiamonds offer great promise for fabricating quantum-enabled devices for physical applications. However, the nanodiamonds need to be suitably combined with a substrate to exploit their properties. Here, we show that ultrathin and flexible glass (thickness 30 microns) can be functionalized by nanodiamonds and nano-shaped using intense femtosecond pulses to design cantilever-based nanomechanical hybrid quantum sensors. Thus fabricated ultrathin glass cantilevers show stable optical, electronic, and magnetic properties of nitrogen-vacancy centers, including well-defined fluorescence with zero-phonon lines and optically detected magnetic resonance (ODMR) near 2.87 GHz. We demonstrate several sensing applications of the fluorescent ultrathin glass cantilever by measuring acoustic pulses, external magnetic field using Zeeman splitting of the NV centers, or CW laser-induced heating by measuring thermal shifting of ODMR lines. This work demonstrates the suitability of the femtosecond-processed fluorescent ultrathin glass as a new versatile substrate for multifunctional quantum devices.

## Introduction

Nitrogen vacancy (NV) centers in diamond nanoparticles have attracted great interest in developing hybrid nanomechanical quantum (HNQ) systems owing to their unique optical, thermal, magnetic, and biological properties with interdisciplinary applications in physics, biology, chemical analysis, and imaging^1–6^. In most HNQ systems, nanodiamonds possessing a single or ensemble of NV centers are coupled with various platforms such as SiN-based resonator, mechanical oscillator, or micro-cantilevers for sensing stress, temperature, and magnetic field using the high sensitivity of NV quantum states to its local environment^7–9^. Additionally, nanodiamonds have been directly injected into various systems for fluorescence imaging and single-cell thermometry with nanoscale resolution^10,11^. The NV centers provide a promising solid-state platform for quantum technologies due to its room temperature operation with long spin coherence times^12,13^ and well-established optical techniques for spin initialization and readout with high fidelity^14,15^. However, there is growing interest in combining NV centers with new materials as well as in developing new fabrication techniques to exploit its potential for quantum sensing.

Previously, NV centers have been prepared on various semiconductor and insulating substrates in the form of cantilevers^16–18^, microcavities^19^, ceramic perovskite^20^, and polymer membranes^21^. Such HNQ devices fabricated on diamond and silicon surfaces have the advantages of being lightweight, and highly responsive to the changes in the local environment. Many potential applications of the NV-based sensors have been demonstrated such as force measurements via magnetic scanning probes and atomic force microscopy (AFM)^22,23^, high-speed actuators^20^ and detection of single molecules and proteins^24–26^. Traditional angle etching technique has been used to fabricate free-standing HNQ systems on the diamond or silicon surfaces using electron-beam-lithography (EBL) and anisotropic plasma etching under high vacuum^16,27,28^. Previously, the interaction of femtosecond laser pulses with NV centers has been studied and these laser pulses have been used to create NV centers on the diamond chip^29,30^.

Recently, high-quality glass with 30 µm thickness having excellent mechanical properties, high flexibility, nanometer surface flatness, and high optical transparency are commercially available. The ultrathin (UT) glass substrate is becoming essential for several mass-market applications owing to its low cost, high surface toughness, and excellent sub-1 nm flatness. UT-glass has been used for a wide range of applications such as fabricating micro-fluidic devices^31–33^, flexible electronic devices^34^, photonics^35,36^, and stable attosecond delay-lines^37^. However, the possibility of combining the extraordinary mechanical properties of UT-glass with the quantum properties of NV center for building flexible quantum sensors remains unexplored. One may wonder, whether the nano-processing capability of femtosecond laser ablation can be used to fabricate nanodiamond enabled UT-glass-based quantum sensing cantilevers.

Here, we demonstrate a femtosecond laser-assisted route to nano-shape nanodiamond functionalized UT-glass in the form of precision cantilevers with nanoscale tips. Our approach proposes to realize hybrid quantum sensors by combining the excellent mechanical properties of UT-glass with the quantum properties of NV centers. We show that a laser-fabricated UT-glass cantilever exhibits NV fluorescence and ODMR which enable quantitatively exploiting these properties for hybrid quantum sensing of acoustic pulses, magnetic field, and laser heating of the cantilever.

## Results

### Laser fabrication of NV-UT cantilevers and its characterization

**Figure 1:**
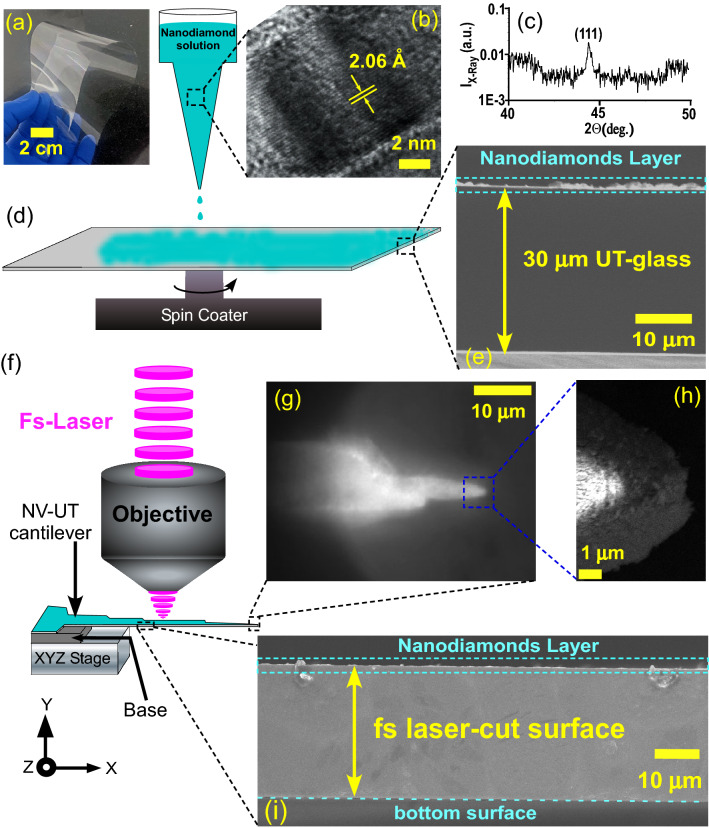
(**a**) Optical image of an UT-glass sheet showing 360° bending (**b**) HRTEM image of a typical single nanodiamond showing atomic lattice spacing of 2.06 Å which corresponded to its (111) plane. (**c**) XRD of the nanodiamonds shows a characteristic peak at 43.9° corresponding to (111) plane. (**d**) Schematic illustration of drop-casting and spin-coating of nanodiamond film on the surface of the UT-glass substrate. (**e**) Cross-section FESEM image of nanodiamond-coated UT-glass for 1 mg/mL concentrated solution. The average thickness of the NV coating is found to be ∼ 1000 nm. (**f**) Experimental setup for fabricating NV-UT cantilevers using femtosecond laser pulses. (**g**, **h**) Brightfield and tilted single tip FESEM image of NV-UT cantilever, respectively. (**i**) Cross-section FESEM image of NV-UT cantilever. A fine laser-cut surface is visible.

We used intense femtosecond laser pulses to cut the UT-glass with nanoscale precision to fabricate cantilevers decorated with nanodiamonds. We begin with a commercially available flexible UT-glass (Fig. 1a) substrate of thickness t = 30 µm and drop cast a sub-micrometer layer of nanodiamonds. The size of the nanodiamonds was about 120 nm and their crystallinity was characterized by high-resolution transmission electron microscopy (HRTEM) and X-ray diffraction (XRD) (Fig. 1b,c). The interplanar spacing as deduced from the HRTEM image is 2.06 Å which corresponds to (111) atomic planes of nanodiamonds^38^. We clearly observed a well-defined characteristic peak at 43.9° which corresponds to (111) atomic lattice planes of the nanodiamond (JCPDS file no. 79-1467)^39^. Nanodiamond colloidal solution (20 µL) of two concentrations (1 mg/mL and 0.5 mg/mL) were spin-coated on UT-glass as shown in Fig. 1d. The coating was dried in a desiccator for about 24 h. The FESEM image of the 1 mg/mL concentrated nanodiamond-coated glass shows a continuous layer of 1000 nm to 2 µm thickness of nanodiamond on top of the glass (Fig. 1e). For lower concentrations of the spin-coated samples the thickness of the nanodiamond layer was found to be 500 nm to 1 µm (Fig. S1). The uniformity of the nanodiamond layer was not controlled, however, they covered the entire surface of the NV-UT cantilever. The fluorescence spectra of the nanodiamonds layer on UT-glass (after laser-cutting) at three different powers (6, 10, and 20 µW) of a green continuous wave laser illumination are shown in Fig. S2a. The characteristic zero-phonon-lines (ZPL) at 575 nm and 637 nm corresponding to NV^0^ and NV^−1^ were observed, respectively, which are superimposed on roughly 100 nm FWHM broadband emission with the peak wavelength near 700 nm. The structure observed near the tail of the spectrum is attributed to calibration artifacts of the spectrophotometer. Additional measurements of the fluorescence spectra at 20 µW excitation power by varying the acquisition time in the spectrophotometer are shown in Fig. S2b. The actual fluorescence counts gradually decrease for lower exposure time while the shape of NV remains preserved.

To fabricate NV-UT cantilevers of chosen dimension, we used a custom-built femtosecond nano-processing setup (Fig. 1f) where the fs pulse train of controllable energy per pulse was focused tightly on the UT-glass. To determine the ablation regime for the glass, we systematically varied the pulse energy and examined the surface for cutting under the microscope. For the pulse energy higher than 0.5 mJ in our setup, the UT-glass cutting was possible with nano-meter resolved edges. The Rayleigh range of the microscope objective was about 15 µm comparable to the thickness of the glass ensuring that by simply raster scanning the fs-beam (scan-speed 1 mm/s) in the desired pattern, the UT-glass was cut out in real-time in the desired shape. As a specific example, we fabricated a few mm long NV-UT cantilevers (Fig. 1g) with a tip of a few hundred nanometers (Fig. 1h). One more example of the tuning-fork-type design is shown in Fig. S3. It is worth mentioning that the fs-pulse produced a local effect without any collateral damage to the fragile glass. Importantly, the laser-glass interaction leaves the nanodiamonds in the immediate vicinity of the laser focus mostly intact (Fig. 1i for NV centers on the laser-cutting edge).

**Figure 2:**
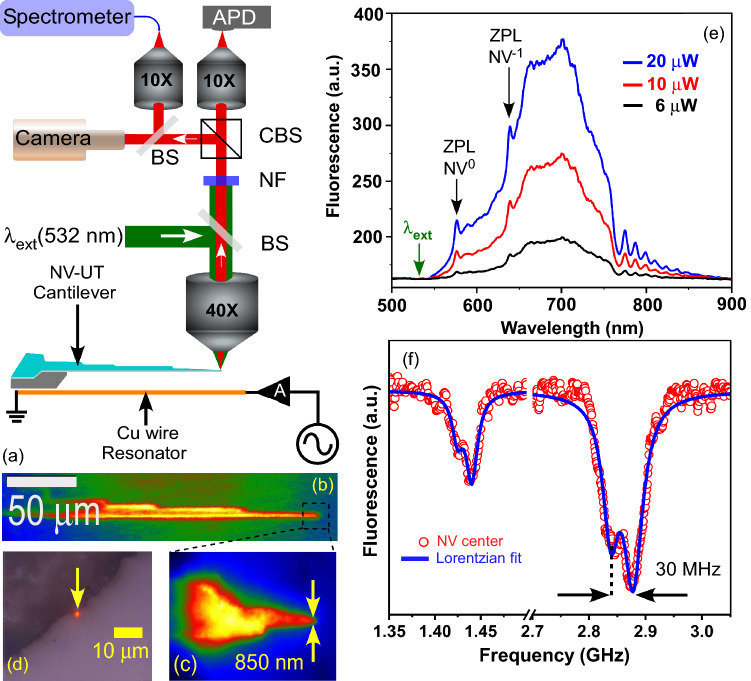
(**a**) Schematic setup for fluorescence imaging, fluorescence spectroscopy, and ODMR spectroscopy. *APD* Avalanche photodiode, *BS* Beam splitter, *CBS* Cube beam splitter, *NF* Notch filter, *A* Amplifier. (**b**) An EMCCD (electron-multiplying CCD) image showing NV fluorescence from the NV-UT cantilever under large-area illumination (green light). (**c**) Magnified fluorescence image of the cantilever tip. (**d**) Fluorescence image of the cantilever under local illumination of the green CW laser. (**e**) Fluorescence spectra of NV center on the cantilever tip under green CW laser (532 nm) excitation (6, 10, and 20 µW) show two zero phonon lines at 575 nm for NV ^0^ and 637 nm for NV^−1^, respectively. (**f**) ODMR spectra were recorded while varying the microwave frequencies from 1.35 to 3.1 GHz. A dip in fluorescence intensity at 1.43 and 2.87 GHz was observed.

It is essential to validate that the NV centers in the nanodiamonds retain their optical, electronic, and magnetic properties following the femtosecond-pulse-based fabrication of the cantilever nano-tip. For this, we build a custom-built optical fluorescence microscope, capable of full-view fluorescence imaging over the entire focal area (using a lamp) as well as localized fluorescence excitation using a laser beam. An external magnetic field and external laser-heating, or acoustic perturbations could also be coupled to the cantilever as desired. Fluorescence images of the cantilever show that the NV fluorescence is all-over the cantilever surface, including its nano-tip (Fig. 2b,c). In addition to this, we also show that it is possible to capture the fluorescence from a micro-spot using laser excitation, for example near the edge of the cantilever, as shown in Fig. 2d. Here, it is worth noting that due to the non-bridging oxygen hole center, the glass substrate itself could emit luminescence^40,41^. We systematically characterized fluorescence due to the intrinsic color centers in the pristine UT-glass (borosilicate) before and after the laser-cutting (without NV coating). The luminescence spectra of pristine UT-glass and femtosecond laser-cut UT-glass are shown in Fig. S4. Please note that these spectra are collected at very high excitation power (5000 µW) and the counts above the noise background are negligible (~ 20). In our NV-coated glass, the fluorescence counts are roughly 100 times larger (~ 1800), for identical measurement parameters. These measurements show that the background from the intrinsic color centers in the UT-glass is negligible when compared to the emission from the NV centers. To show that the NV centers on the cantilever tip preserved their quantum properties, we measured the fluorescence spectra of the nanodiamonds under green CW laser (532 nm) excitation of aforementioned powers (6, 10, and 20 µW) at the tip of the NV-UT cantilever, as shown in Fig. 2e. The optical emission spectra show the characteristic ZPL at 575 nm and 637 nm corresponding to NV^0^ and NV^−1^, respectively, which confirms the changeless quantum properties.

Furthermore, we performed optical detection of magnetic resonance (ODMR), a characteristic quantum property of the NV centers, by collecting laser-induced fluorescence from the NV-UT cantilever tip. The NV-UT cantilever was simultaneously excited by a microwave magnetic field, whose frequency was scanned from 1.35 to 3.0 GHz and the fluorescence was detected with an avalanche photodiode (APD). The APD signal showed clear dips in the total fluorescence yield repeatably near the expected ODMR lines at 1.44 and 2.87 GHz. As shown in Fig. 2f, the experimental data were fitted with a double Lorentzian with resonance frequency splitting of 30 MHz (FWHM of 83 MHz) at 2.87 GHz, which is consistent with previous such measurements^42^. Additionally, the zero-field splitting of NV lines was also observed previously and its origin is attributed to internal stress in the nanodiamonds. The mechanism of MW modulation of the fluorescence in NV centers is attributed to its specific atomic structure and has been discussed in several previous works^43,44^. In the following, we shall exploit these properties of NV centers for demonstrating hybrid quantum sensing applications of the glass cantilever.

### Hybrid quantum sensing applications

**Figure 3:**
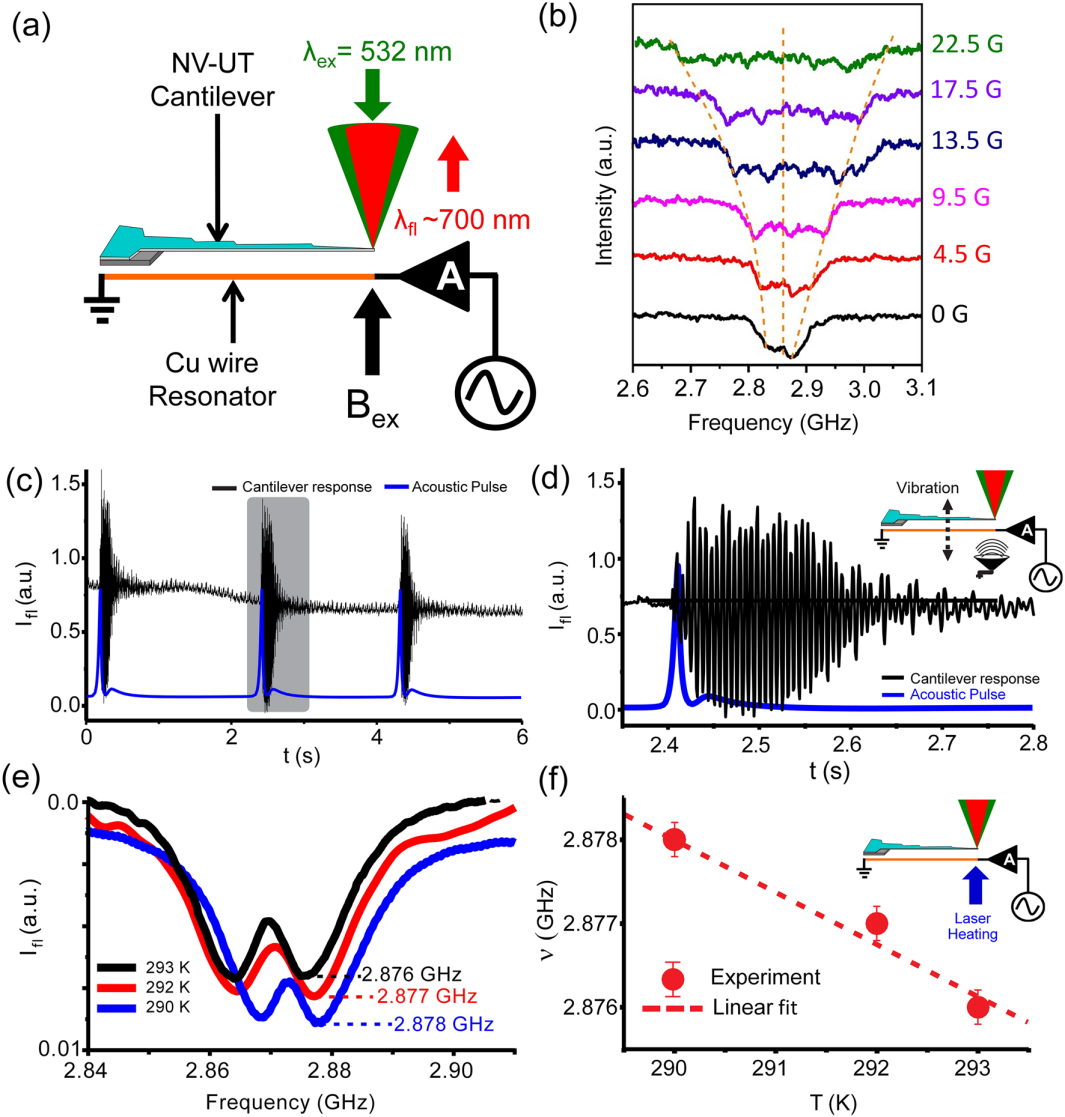
(**a**) Schematic of the experimental setup for Zeeman splitting. (**b**) ODMR spectra with and without external applied magnetic field B_ex_ = 0, 4.5, 9.5, 13.5, 17.5, and 22.5 G. The broadening of spectra is caused by a gradual increase in B_ex_ (**c**) Open-end nanotip NV-UT cantilever response to a single acoustic pulse of 160 Hz. (**d**) Zoom of the NV-UT cantilever response and the inset shows a schematic of the experimental setup where a speaker generates the acoustic pulse at the Normal axis (**e**) Shift in ODMR signal as a function of temperature change (**f**) Shift in resonance frequency as a function of temperature was found to be δν/δT = 1.3 MHz/K and inset shows the schematic of experimental setup where the temperature was varied near the tip using a 445 nm CW laser.

Figure 3a demonstrates schematics of the magnetometry setup based on the Zeeman splitting of the ODMR spectra of the NV center. As shown in the data of Fig. 3b, the ODMR, we observed Zeeman splitting of the ODMR lines with an increase in the external magnetic field (B_ex_). The B_ex_ was varied from zero to 22.5 G. Additionally, the ODMR lines gradually dropped and broadened with B_ex_. The overall profile of Zeeman splitting agrees with previously reported magneto-mechanical sensors^45,46^.

The sensitivity of the magnetic field was 20 ± 2 MHz/Gauss which follows the Zeeman splitting formula for ∆f = 2γB_ex_, where ∆f is frequency broadening and γ = 2.8 MHz/Gauss^47^. In these measurements, the magnetic field was mostly homogeneous within the tip volume.

To measure the acoustic vibrations with the NV-UT cantilever, we exploit a modulation of the tip-fluorescence when the NV-UT cantilever is displaced from the exact focus due to sound pressure. Calibration between the change in fluorescence and tip displacement is shown in Fig. S5 which suggests that the NV-UT cantilever position can be linearly sensed. For acoustic measurements, we carefully placed the NV-UT cantilever and excited it in ambient condition with an acoustic impulse. A typical nano-mechanical response of the NV-UT cantilever is shown in (Fig. 3c,d) where the quality factor of the vibration was around 40 in the air. The performance was repeated by three impulses which shows that the NV-UT cantilever can serve as an acoustic sensor.

Lastly, we show that the NV-UT cantilever can directly measure laser-heating of the tip with microkelvin resolution. The NV thermometry is well established and exploits the shift in ODMR of the NV ensemble (Fig. 3e) with respect to the temperature change. CW blue laser (445 nm) is used to heat the cantilever tip. The thermal effect of the excitation wavelength (532 nm) is negligible since its power is low. The absence of heating by 532 nm laser is further confirmed by a negligible shift in ODMR spectra taken at different power of 6, 10, and 20 µW (Fig. S6). In our case, by varying the blue laser power, we observed that the ODMR resonance shifts with 1.3 MHz/K^48^, as shown in Fig. 3f, suggesting that this tip can be used to sense the local temperature of the environment.

## Discussion

The possibility of combining nanodiamonds and UT-glass with femtosecond laser-based fabrication offers flexibility to design versatile devices. One can fabricate any desired shape and size of the cantilever where its resonant frequency can be geometrically tuned with our simple fabrication approach. Furthermore, the laser-processing should allow using even thinner glass to fabricate cantilevers, such as the one prepared by thermal stretching down to about ≤ 3 µm thickness^49^. Moreover, in our approach, the entire cantilever is deposited with nanodiamonds which could allow fluorescence detection at multiple places for specific applications. Our measurements correspond to an ensemble of NV centers in 120 nm nanodiamonds. It may be worth exploring whether this approach allows the measurement of a single NV center by using sub-10 nm size nanodiamonds. Although, with the femtosecond-based approach it is difficult to place a single nanodiamond at the nanotip in a controlled fashion and such an objective can be met by coupling glass cantilevers with optical trapping. In addition, it is also possible that these tiny NV-UT cantilevers can be cooled to cryogenic temperatures for low-temperature measurements.

In conclusion, we demonstrate that the UT-glass functionalized with nanodiamonds containing NV centers can be laser-cut in desired shapes with nanoscale precision for fabricating hybrid nanomechanical cantilevers with quantum sensing capabilities. We demonstrate that the NV-UT cantilever allows the sensing of acoustic, thermal, and magnetic fields quantitatively. We envisage that laser-processed fluorescence UT-glass devices can be coupled with optical cavities for various precision optomechanical experimental tests. This could lead to the mass production of affordable cantilevers for multifunctional imaging and sensing applications.

## Methods

### Materials

Ultrathin glass sheets were obtained from Schott glass. The glass has a thickness t = 30 µm, young’s modulus 72.9 kN/mm^2^, bending radius < 1 mm, and excellent surface roughness (< 1 nm). The nanodiamonds (Nitrogen vacancy 3 ppm, 120 nm average particle size, 1 mg/mL in deionized water) colloidal solution was purchased from Sigma-Aldrich and was used without further purification and dilution.

### Fabrication of NV-UT cantilever

The nanodiamond colloidal solution was spin-coated at 3000 rpm for 20 s on an ultrathin glass sheet (76 × 26 mm^2^) having t = 30 µm. In spin coating, 20 µL colloidal solution of different nanodiamonds concentrations (1 mg/mL and 0.5 mg/mL) was spread evenly over the whole surface of the substrates. Before thin film fabrication, the substrate was pre-cleaned in acetone and methanol using an ultrasonic bath and then dried using Nitrogen gas. The nanodiamond-coated ultrathin glass was dried in a desiccator overnight. A commercial laser system (FEMTOLASERS, Austria) produced fs-pulses having 25 fs pulse duration at λ_IR_ = 800 nm central wavelength at 1 kHz rep rate with 1–2 mJ energy per pulse. Intense fs pulses were tightly focused by a 10X (0.25 NA) objective on a nanodiamond-coated ultrathin glass sheet surface. The estimated peak intensity at the focus was varied by ND filter and could reach 9 × 10^18^ W/cm^2^. The sample stage was automated and offered nanometer precision to fabricate cantilevers of desired design at ambient conditions^50^. The exposure time of fs-pulses was varied from 1 to 1000 ms raster scanning the ultrathin glass (velocity: 1–100 mm/s, XYZ–scanning stage, Thorlabs, USA). The automation program is written in LabView.

### Fluorescence imaging, spectroscopy, and ODMR setup

We custom design a fluorescence microscope cum ODMR spectroscopy setup (Fig. 2a). For fluorescence imaging, we used a continuous-wave (CW) solid-state green laser (λ_ex_ = 532 nm) whose power was adjusted from 10 µW to 2 mW using another ND filter (ND4). To capture a high-resolution fluorescence image of the NV-UT cantilever under low-light conditions, an EMCCD (Andor) cooled to − 80 °C was used while a colored fluorescence image was captured by a colored ThorLab cooled camera (24-bit). A fluorescence notch filter (OD-6, ∆λ = 10 nm) was used to remove the 532 nm excitation. A single Cu wire (radius = 10 µm) on a printed circuit board was used as a resonator^51^. A signal generator (Rohde & Schwarz, SMB 100 A) was used to feed a microwave signal (1 GHz to 3.2 GHz with a step size of 1 MHz). An amplifier (Mini Circuits, ZHL-42W+) was used for a 30 dB gain. A 40X objective (ZEISS, 0.75 NA) was used to maximize the collection efficiency of the fluorescence photons. All the experiments were performed five times.

### HNQ sensor setup

The aforementioned fluorescence microscope cum ODMR spectroscopy setup was further modified to study the effect of the magnetic field, acoustic vibrations, and temperature. An external magnetic field was produced by a solenoid made with a Cu wire (radius = 1 mm) solenoid (length = 4 cm) was placed under the resonator. Gauss meter’s probe was placed about 10 µm above the resonator wire. For the temperature HNQ sensor, the temperature was varied near the NV-UT cantilever tip using a CW blue laser (445 nm).

## Supplementary Information

Supplementary Figures.

## Data Availability

The data/code generated in this study can be made available by KPS (kpsingh@iisermohali.ac.in) upon reasonable request. The corresponding author is responsible for submitting a competing interests statement on behalf of all authors of the paper. This statement must be included in the submitted article file.
