# Supplementary material for: Nanodiamonds enable femtosecond-processed ultrathin glass as a hybrid quantum sensor
Source: Sci Rep. 2023 Apr 18;13:6286. doi: 10.1038/s41598-023-30689-7 (PMC10113221; doi:10.1038/s41598-023-30689-7)
Supplement: Supplementary file 1 — Supplementary Figures. [file 41598_2023_30689_MOESM1_ESM.pdf]

Nanodiamonds enable femtosecond-processed ultrathin glass as a hybrid quantum sensor

Bhavesh K. Dadhich[1], Biswajit Panda[1], Mehra S. Sidhu[1], and Kamal P. Singh[1,*]

[1]Indian Institute of Science Education and Research Mohali, Sector 81, Mohali, 140306, India
[*]kpsingh@iisermohali.ac.in

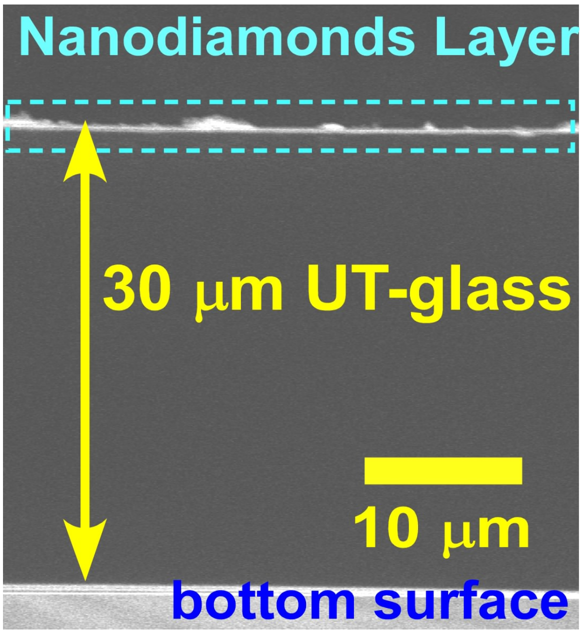

Figure S1. Cross-section FESEM image of nanodiamond-coated ultrathin glass for 0.5 mg/mL concentrated solution. The average thickness of the NV coating is found to be $\sim$ 500 nm.

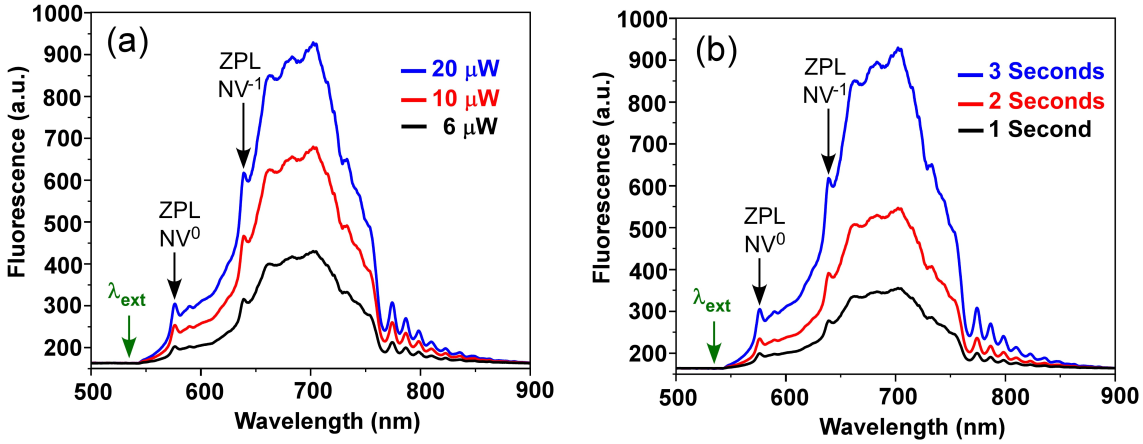

Figure S2. (a) Fluorescence spectra of NV centers before fs-pulse exposure under green CW laser (532 nm) excitation (6, 10, and 20 μW). (b) Effect of exposure time on the fluorescence spectra of NV center before fs-laser exposure under green CW laser (532 nm) excitation (20 μW).

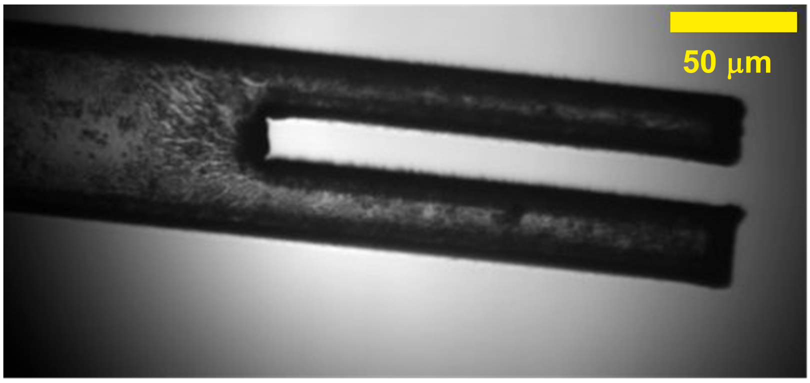

Figure S3. Brightfield image of tuning fork type ultrathin glass cantilever.

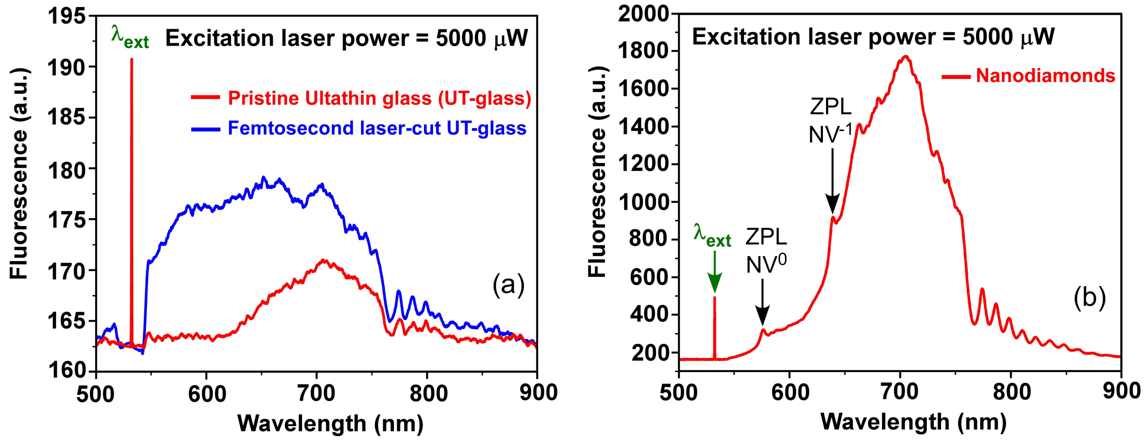

Figure S4. Comparison of fluorescence in pristine ultrathin glass, laser-cut glass, and NV-coated glass. (a) Emission spectra of the ultrathin glass before and after fs laser exposure at the excitation power (5000 μm). An edge near the 532 nm is due to the notch filter. (b) Emission spectra of the nanodiamonds at the same excitation power (5000 μm).

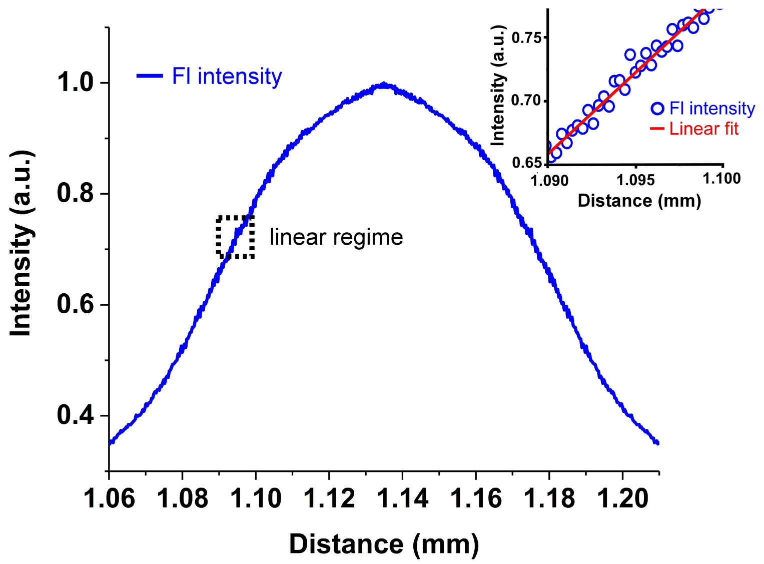

Figure S5. Calibration of fluorescence intensity by the gradual transition of 40X objective from the cantilever tip. For acoustic HQD experiments were performed in a linear regime (inset).

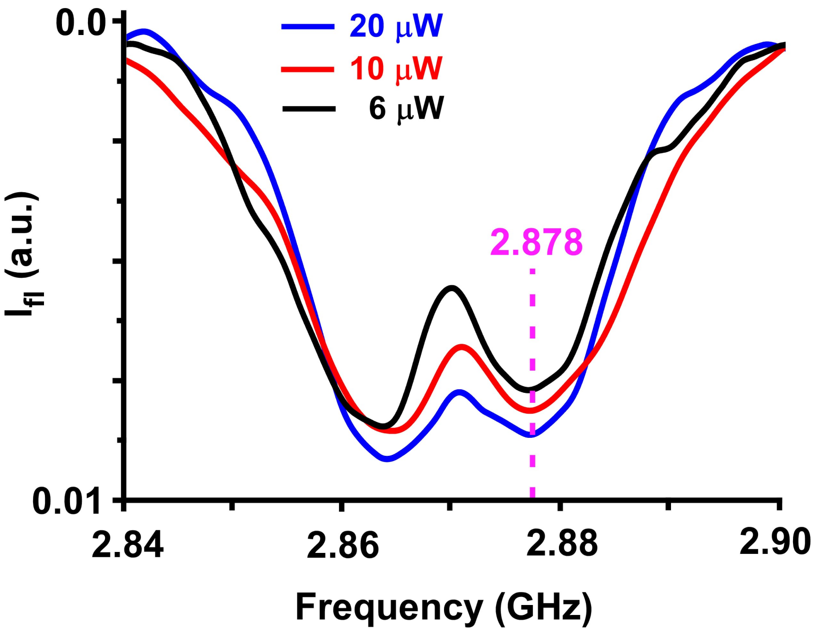

Figure S6. ODMR spectra at different power (6, 10, and 20 μW) of excitation wavelength. Here, other excremental parameters were kept the same as in figure 3(e), except the blue laser is off.
